# Molecular Characterization of Staphylococci Recovered from Hospital Personnel and Frequently Touched Surfaces in Tianjin, China

**DOI:** 10.1155/2022/1061387

**Published:** 2022-08-10

**Authors:** Yuting Liu, Liqin Chen, Yuping Duan, Zhen Xu

**Affiliations:** ^1^Tianjin Xiqing Hospital, Tianjin 300380, China; ^2^Department of Sanitary Toxicology and Chemistry, Tianjin Medical University, Tianjin 300070, China; ^3^Tianjin Key Laboratory of Environment Nutrition and Public Health, Tianjin Medical University, Tianjin 300070, China; ^4^Center for International Collaborative Research on Environment Nutrition and Public Health, Tianjin Medical University, Tianjin 300070, China

## Abstract

Staphylococci are major hospital-associated pathogens, and the dissemination of methicillin-resistant staphylococci in hospitals poses a great challenge for managing hospital-acquired infections. Little is known about the dissemination of staphylococci recovered from the hospital environment and personnel in China. In this study, antimicrobial susceptibility tests, *mecA* gene detection, SCC*mec* typing, and multilocus sequence typing (MLST) were performed to clarify the molecular epidemiology of staphylococci in a large hospital in Tianjin, China. One hundred and ninety-five staphylococci were recovered, and 94% of isolates were resistant to at least one antibiotic. Eighty-five staphylococci were *mecA* gene-positive, and 40% of them harbored SCC*mec* IV and V. The genotype of *Staphylococcus aureus* (*S. aureus*) was ST25, and the dominant genotype of methicillin-resistant *Staphylococcus epidermidis* (MRSE) was ST59. Three new sequence types were assigned as ST840, ST841, and ST842. One (2%) frequently touched surface was contaminated by *S. aureus*, which suggested that environmental contamination occurred in the hospital in China. Nineteen (39%) frequently touched surfaces were contaminated by methicillin-resistant coagulase-negative staphylococci (MRCoNS), and 46% of HP carried MRCoNS. Varied staphylococcal species and antimicrobial-resistance rates were observed between staphylococci that were recovered from hospital personnel and frequently touched surfaces. The transmission of MRSE and *S. aureus* between hospital personnel and frequently touched surfaces was detected. Hospital items and personnel may act as reservoirs of antimicrobial-resistant staphylococci, and cleaning strategies should be carried out to decrease the dissemination of antimicrobial-resistant staphylococci in hospitals in China.

## 1. Introduction

*Staphylococcus aureus* (*S. aureus*) and coagulase-negative staphylococci (CoNS) pose a health threat to humans worldwide. In China, staphylococci (13.4%) are on third place as a cause of clinical infection [1]. The majority (65.5%) of bloodstream infections were caused by staphylococci in a tertiary hospital in Beijing [2] and staphylococci were the most common Gram-positive cocci that were recovered from the clinic in Shanghai [3]. The clinical significance of staphylococcal species cannot be ignored. Meanwhile, studies confirm that the frequently touched surfaces (FTS) within the hospital environments might be potential reservoirs of nosocomial pathogens, and the hands of hospital personnel (HP) might be contaminated by touching the contaminated environment surfaces [4]. Afterward, HP may transfer pathogens to vulnerable patients, or pathogens may be transmitted directly from contaminated surfaces to vulnerable patients [5].

*S. aureus* contamination on frequently touched surfaces within the hospital environment was observed worldwide [4]. In South Africa, the *S. aureus* contamination rate on the frequently touched surface was 58.7%, and 9.5% were contaminated by *S. aureus* in the United States [4]. Moreover, 18% and 7.9% of the FTS were contaminated by *S. aureus* in Asia and Europe, respectively [4]. The *S. aureus* contamination rates on FTS in South Africa are significantly higher than in other continents. In Taiwan, the *S. aureus* contamination rate on the FTS was 6%, and 1.1% were contaminated by MRSA [6]. Unfortunately, no data are available to know the contamination rates of *S. aureus* and CoNS on FTS in mainland China, and the phylogenetic relationship between staphylococci that was recovered from HP and FTS remains unknown.

The clinical significance of *S. aureus* and CoNS has been widely recognized, and it can increase significant morbidity, mortality, and healthcare costs [4, 7]. The dissemination of antimicrobial-resistant staphylococci in hospital personnel and the environment poses a great health threat to the vulnerable patient population. Therefore, it is necessary to understand the dissemination of antimicrobial-resistant staphylococci in healthcare settings. HP and FTS may act as reservoirs of antimicrobial-resistant pathogens. Therefore, this study was performed to understand the diversity, antimicrobial resistance, and genotype of *S. aureus* and CoNS that were recovered from hospital frequently surface and HP of a tertiary hospital in Tianjin, China.

## 2. Materials and Methods

### 2.1. Sampling and Isolation

The research protocol and informed consent were approved by the Ethics Committee of Tianjin Xiqing Hospital (approval No TMUaMEC2017017 and xqyyll-2021-17). Verbal permission was acquired from all participants before sampling.

The hospital is a large (1400 bed and 1258 hospital staff) teaching hospital that handles about 2500 patients per day and is located in the north of Tianjin, China. The collection date was from September to December in 2018. Hospital personnel was from the Chinese medicine department, dermatology department, endocrinology department, emergency room, ultrasound medicine, medical exam center, orthopedics department, proctology department, rehabilitation department, and thoracic surgery department (Table S1). Frequently touched surfaces included water tap, door handles, medical equipment, checking bed, supporter, and registration machine of 19 departments (Table S2). The personnel were trained by practical demonstration before sample collection. Sterilized cotton dry swabs (*n* = 128) were moistured with sterilized saline and then used to collect hand or nasal samples of HP and frequently touched surface samples randomly, and the samples were transferred back to the lab within 2 hours with sterilized saline. The swabs were plated on mannitol salt agar and incubated at 37°C for 24–48 hours. The single colony with different morphology was inoculated and purified on nutrient agar (OXOID, UK) and incubated at 37°C for 24 hours.

### 2.2. Identification

Staphylococci isolated from FTS were identified by matrix-assisted laser desorption/ionization time of flight mass spectrometry (MALDI-TOF MS) in a positive linear mode (2000–20,000 *m*/*z* range) as described previously [8]. The resulting spectra were compared with reference spectra using the Biotyper 3.0 software (Bruker Daltonics, Germany). *Escherichia coli* DH5*α* (Bruker Daltonics, Germany) was used as a standard for calibration and quality control. 16S rRNA genes of isolates recovered from HP were amplified and sequenced by Sangon Biotech (Shanghai Ltd.). The sequences were then compared with the NCBI database [9].

### 2.3. Antimicrobial Susceptibility Test

The disc diffusion method was used to test the antimicrobial susceptibility of staphylococci that were recovered from the hospital environment and personnel, and a panel of 11 antibiotics, including chloramphenicol (C, 30 *µ*g), clindamycin (CD, 2 *μ*g), gentamicin (CN, 10 *µ*g), ceftaroline (CPT, 30 *µ*g), erythromycin (E, 15 *µ*g), cefoxitin (FOX, 30 *µ*g), levofloxacin (LEV, 5 *µ*g), linezolid (LZD, 30 *µ*g), penicillin (PG, 10 units), tetracycline (T, 30 *µ*g), teicoplanin (TEC, 30 *µ*g), were included. The interpretation of susceptible, intermediately-resistant, or resistant was determined by the CLSI [10].

### 2.4. *mecA* Gene Detection and SCCmec Typing

mA1 and mA2 primers were used to amplify the *mecA* gene, and eight pairs of primers were used for amplification of class A, B, and C *mec* complex and the *ccr* 1–5 complex. The PCR products were visualized by gel electrophoresis. The combination of the *mec* gene complex and the *ccr* gene complex was used to determine the SCC*mec* types [11]. Sequences of primers and amplification protocols were referred to Kondo et al. [12].

### 2.5. Multilocus Sequence Typing of *S. aureus* and *S. epidermidis*

*S. aureus* and *S. epidermidis* were the most common staphylococcal species that were identified in clinics in China (https://www.carss.cn), and thus, *S. aureus* and *S. epidermidis* were selected for MLST typing. Housekeeping genes of *S. aureus* and *S. epidermidis* were amplified according to the standard guidelines [13, 14] and sequenced by Sangon Biotech (Shanghai, China). The sequence type (ST) was assigned by the MLST database (https://pubmlst.org/).

### 2.6. Statistical Analysis

The *χ*^2^ test was used to analyze the quantitative variables. A *P* value <0.05 was considered statistically significant.

## 3. Results

### 3.1. Staphylococcal Isolates

Seventy-nine samples were collected from the hands or noses of 79 hospital personnel, and 49 samples were collected randomly from frequently touched surfaces. One hundred and ninety-five staphylococci were recovered, including 109 isolates recovered from HP, and 86 were isolated from FTS of the hospital. *S. epidermidis* (*n* = 72) was the dominant species that was recovered from HP, followed by *S. hominis* (*n* = 27). Only 1 *S. capitis* and 1 *S. pasteuri* were identified in HP samples. The main species that were isolated from FTS were *S. epidermidis* (*n* = 27), followed by *S. hominis* (*n* = 13) and *S. capitis* (*n* = 13). In contrast, only 1 *S. aureus*, 1 *S. pettenkoferi*, and 1 *S. succinus* were recovered from FTS samples.

### 3.2. Antimicrobial Susceptibility Profile

Antimicrobial susceptibility profiles of 195 staphylococcal isolates are listed in Table 1. Staphylococcal isolates showed the highest resistance rate (67%) towards penicillin and the lowest resistance rate (3%) towards linezolid. The rest 9 antibiotics were ordered by their resistant rate as follows: erythromycin (*n* = 114 (58%) > cefoxitin (*n* = 85 (44%) > levofloxacin (*n* = 21 (24%) > clindamycin (*n* = 35 (19%) > tetracycline (*n* = 33 (17%) > gentamicin (*n* = 30 (15%) > chloramphenicol (*n* = 28 (14%) > teicoplanin (*n* = 24 (12%) > ceftaroline (*n* = 7 (4%) (Tables 1, S1, and S2).

### 3.3. *mecA* Gene Detection and SCCmec Typing

Eighty-five staphylococcal isolates harbored the *mecA* gene, including 55 (50.5%) isolates that were recovered from HP and 30 (35.3%) isolates that were isolated from FTS. The SCC*mec* type V (*n* = 16, 29%), I (*n* = 9, 16%), and II (*n* = 9, 16%) were mainly identified in MRCoNS that were recovered from HP, and SCC*mec* IV (*n* = 7, 23%) and V (*n* = 6, 20%) were the dominant types that were found in MRCoNS isolated from FTS. The SCC*mec* types were not determined in 10 isolates due to the lack of either the *mec* complex or the *ccr* complex (Tables 2, S1, and S2).

### 3.4. Multilocus Sequence Typing of *S. aureus* and *S. epidermidis*

One *S. aureus* recovered from FTS was determined to be ST25, and 44 out of 57 *S. epidermidis* recovered from HP and FTS belonged to clonal complex 2 (CC2). Four *S. epidermidis* belonged to minor CCs, and 6 *S. epidermidis* were categorized into other CCs. In addition, three new STs were determined, including ST840 (*n* = 1), ST841 (*n* = 1), and ST842 (*n* = 1) (Tables 3, S1 and S2).

## 4. Discussion

Few data are available to understand the contamination rate of *S. aureus* and MRCoNS on FTS in hospitals in mainland China, and the phylogenetic relationship of staphylococci that was recovered from HP and FTS remains unknown. In this study, we had an insight into the genotypes of staphylococci that were recovered from FTS and HP.

*S. capitis, S. epidermidis*, and *S. hominis* are species that are frequently recovered from the body surface of humans [7]. In this study, *S. epidermidis* and *S. hominis* were the main species that were recovered from HP. In contrast, *S. capitis*, *S. epidermidis*, and *S. hominis* were the major species that were recovered from FTS. It is suggested that the FTS was significantly affected by human microbiota, and surface cleaning should be carried out from time to time to decrease the transmission of antimicrobial-resistant bacteria. The colonization rate of *S. epidermidis* among HP was significantly higher than that of FTS (*P* < 0.000003124), and no significant difference was observed in other staphylococcal species. *S. equorum, S. haemolyticus, S. pettenkoferi, S. saprophyticus*, and *S. succinus* were exclusively found in FTS, which suggested that FTS might be contaminated by diverse sources. In this study, 1 out of 49 (2%) FTS samples was contaminated by *S. aureus*, which was lower than the *S. aureus* contamination rate on the frequently touched surface in Taiwan and other countries [4, 6]. Fifteen out of 79 (19%) HP carried *S. aureus*, which was cited from published work [9]. Diverse staphylococcal species were found in samples collected from FTS, and attention should be paid to hospital environment monitoring and cleaning.

In addition, CoNS was detected in 75.5% (37/49) samples of FTS, and 39% (19/49) of FTS were contaminated by MRCoNS. In this study, 46% of HP carried MRCoNS, which was higher than the rate of the international report [15]. Thus, hand hygiene practices should be emphasized by hospitals in China. Four (4%) staphylococci that were recovered from HP were fully susceptible to all tested antibiotics, while 11 (13%) isolates that were recovered from FTS were fully susceptible to all tested antibiotics. For isolates recovered from HP, the resistance rate of clindamycin, tetracycline, gentamicin, and linezolid was lower than in previous international surveillance reports [15]. In contrast, the resistance rate of penicillin, erythromycin, cefoxitin, and levofloxacin was higher than in the previous report [15]. The resistance rates of isolates that were recovered from FTS towards gentamicin were much higher than that of isolates recovered from HP (*χ*^2^ = 16.852, *P* < 0.05). In contrast, the resistance rates of isolates that were recovered from HP towards erythromycin (*χ*^2^ = 5.1809, *P* < 0.05), cefoxitin (*χ*^2^ = 4.1302, *P* < 0.05), and penicillin (*χ*^2^ = 33.203, *P* < 0.05) were significantly higher than that of isolates recovered from FTS (Table 3). Multiresistant CoNS may limit the medicinal options and aggravate treatment strategies [16], and the wide dissemination of multiple-resistant staphylococci in HP and FTS was a worrisome finding.

*mecA* gene encodes penicillin-binding protein 2a, which confers methicillin resistance [17]. The carriage rate (50.5%) of the *mecA* gene in staphylococci that were recovered from HP was significantly higher than the rate (35.3%) of staphylococci that were isolated from FTS (*χ*^2^ = 4.1302, *P* < 0.05). MRCoNS may act as concealed reservoirs for antibiotic resistance and virulence genes [18], and the high prevalence of MRCoNS in this study is an alerting finding. *mecA* gene is located on a mobile genetic element, which is named staphylococcal cassette chromosome *mec*. The SCC*mec* types I, II, and III were hospital-associated and SCC*mec* IV and V were community-associated [19]. The SCC*mec* types I, II, IV, and V were identified in *S. epidermidis* that were recovered from HP and FTS. In contrast, SCC*mec* VI was exclusively identified in *S. epidermidis* that were recovered from FTS, and SCC*mec* I and IX were exclusively detected in *S. epidermidis* that were isolated from HP. The SCC*mec* types VI and IX were first identified in staphylococci that were recovered from Portugal and Thailand, respectively [11]. In this study, the majority of staphylococci that were recovered from HP and FTS harbored community-associated SCC*mec* IV and V; however, it is worth noting that hospital-associated SCC*mec* I and II were mainly identified in staphylococci that were recovered from HP (Table 2). It is reported that SCC*mec* IV and V were higher than SCC*mec* I, II, and III due to their smaller size [20], and the difference in dominant SCC*mec* identified in staphylococci that were recovered from HP and FTS may be due to their varied transmissible abilities. ST25 was identified in *S. aureus* that was recovered from HP and FTS (Table 3) [9], which suggested the cross-transmission of *S. aureus* between HP and FTS.

*S. epidermidis* ST59, ST200, and ST210 were recovered from samples that were collected from the hands of HP as well as FTS, which suggested the transmission between the hands of HP and FTS in the hospital. *S. epidermidis* ST200 was reported to be associated with healthy individuals only, and ST210 was the genotype that was previously recovered from the hands of HP [21]. Therefore, the FTS of the hospital might be influenced by the microbiota of both HP and healthy individuals. *S. epidermidis* ST59 was the community-associated pathogenic clone in Asia [22], and the isolation of *S. epidermidis* ST59 in this study indicates that the HP and FTS were contaminated by pathogenic clones. Hospital environment screening should be included in regulatory monitoring.

In this study, 32 sequence types were determined in *S. epidermidis*, and ST59, ST200, and ST210 were found in *S. epidermidis* that were recovered from samples of HP and FTS. In contrast, 12 sequence types were exclusively identified in FTS, and 17 sequence types were only identified in HP. However, most STs belonged to CC2, which is the dominant clonal complex in HP in China [21]. Thus, it is suggested that CC2 was also the dominant clone that disseminated in the hospital environment. It is worth noting that *S. epidermidis* ST210 was identified in samples that were collected from HP and FTS, respectively, and the samples were both gathered from the emergency room. Thus, the cross-transmission of *S. epidermidis* between HP and FTS was highly plausible. Microbiota of patients might affect the microbiota of FTS [21] and thus might explain the inconsistency of ST between staphylococci that were recovered from HP and FTS. No data were collected from patients which was a limitation of this study. ST5, ST17, ST20, ST89, ST152, ST173, ST192, ST218, ST235, ST251, and ST291 were previously identified in staphylococci that were recovered from HP in Shanghai [21]. Consistent with the previous study, ST5, ST20, ST89, ST152, ST173, ST192, ST235, ST251, and ST291 were detected in HP of our study, while ST17 and ST218 were identified in FTS only. Moreover, ST35, ST247, ST249, ST454, ST840, ST841, and ST842 that were identified in HP of this study were not reported by the previous study [21]. ST6 was reported to be identified in patients [21], which was detected in the FTS of this study. In addition, some STs of *S. epidermidis* that were isolated from FTS, such as ST4, ST325, ST337, ST344, ST374, ST788, ST826, ST878, and ST916, were not reported before. Diverse STs were identified in *S. epidermidis* that were recovered from the hospital environment, and attention should be paid to hospital environment monitoring.

## 5. Conclusions

In conclusion, species of staphylococci that were isolated from FTS were much more diverse than that from HP. The antimicrobial-resistance rate, *mecA*-genes carriage rate, and the sequence types differed between staphylococci recovered from HP and FTS. *S. epidermidis* STs identified in HP samples were associated with both community and hospital clones, while STs of *S. epidermidis* that were isolated from FTS were partially associated with hospital clones. The sources of some *S. epidermidis* isolated from FTS remain unknown, and attention should be paid to tracing the sources of clones that were disseminated in the hospital environment in China. *S. aureus* ST25 was identified in samples of HP and FTS, which suggested the transmission of *S. aureus* between HP and FTS. Three new MLST types were identified in this study. The limitation of this study was that no patients were recruited in this study; whereas, the cross-transmission of *S. aureus* and *S. epidermidis* between HP and FTS in the hospital was observed, and cleaning strategies should be carried out to decrease the dissemination of antimicrobial-resistant staphylococci in hospitals in China.

## Figures and Tables

**Table 1:** Antimicrobial susceptibility profile of *mecA*-positive and negative staphylococci that were recovered from hospital personnel and frequently touched surfaces.

Antibiotics	Interpret	*mecA*-positive staphylococci		*mecA*-negative staphylococci	
		HP (*n* = 55)	FTS (*n* = 30)	HP (*n* = 54)	FTS (*n* = 56)
C (30 *µ*g)	R	14	3	2	9
	I	1	2	2	3
	S	40	25	50	44

CD (2 *μ*g)	R	14	3	6	12
	I	19	4	18	15
	S	22	23	30	29

CN (10 *µ*g)	R	4	10	2	14
	I	3	0	1	2
	S	48	20	51	40

CPT (30 *µ*g)	R	3	0	1	3
	I	2	4	1	3
	S	50	26	52	50

E (15 *µ*g)	R	38	16	34	26
	I	9	6	11	9
	S	8	8	9	21

FOX (30 *µ*g)	R	55	30	0	0
	S	0	0	54	56

LEV (5 *µ*g)	R	13	4	2	2
	I	4	3	0	1
	S	38	23	52	53

LZD (30 *µ*g)	R	0	3	0	2
	S	55	27	54	54

PG (10 unit)	R	47	18	45	20
	S	8	12	9	36

T (30 *µ*g)	R	11	3	12	7
	I	0	0	1	1
	S	44	27	41	48

TEC (30 *µ*g)	R	3	2	9	10
	I	28	6	16	23
	S	24	22	29	23

*Note.* HP, hospital personnel; FTS, frequently touched surfaces; C, chloramphenicol; CD, clindamycin; CN, gentamicin; CPT, ceftaroline; E, erythromycin; FOX, cefoxitin; LEV, levofloxacin; LZD, linezolid; PG, penicillin; T, tetracycline; TEC, teicoplanin.

**Table 2:** SCC*mec* elements determined in hospital personnel and frequently touched surfaces.

Source	SCC*mec* types													
	I	II	IV	V	VI	IX	A/1	A/5	B/5	C/1	C/2	SCC	Pseudo (*ψ*) SCC	Pseudo (*ψ*) SCC*mec*
HP (*n* = 55)	9 (16%)	9 (16%)	6 (11%)	16 (29%)	0 (0)	2 (4%)	3 (5%)	2 (4%)	2 (4%)	0 (0)	2 (4%)	3 (5%)	1 (2%)	0 (0)
FTS (*n* = 30)	1 (3%)	3 (10%)	7 (23%)	6 (20%)	1 (3%)	0 (0)	1 (3%)	1 (3%)	1 (3%)	1 (3%)	2 (4%)	4 (13%)	1 (3%)	1 (3%)

*Note.* HP, hospital personnel; FTS, frequently touched surface.

**Table 3:** Comparative profiling of staphylococcal species, antimicrobial resistance, *mecA* gene carriage, and STs of staphylococci recovered from hospital personnel and frequently touched surfaces.

Staphylococcal			HP	FTS	*P* value
Species			*S. aureus * ^†^	*S. aureus*	—
			*S. capitis*	*S. capitis*	
			*S. cohnii*	*S. cohnii*	
			*S. epidermidis*	*S. epidermidis*	
			—	*S. equorum*	
			—	*S. haemolyticus*	
			*S. hominis*	*S. hominis*	
			*S. pasteuri*	*S. pasteuri*	
			—	*S. pettenkoferi*	
			—	*S. saprophyticus*	
			—	*S. succinus*	
			*S.warneri*	*S. warneri*	

Antimicrobial-resistance rate	C		15%	14%	>0.05
	CD		18%	17%	>0.05
	CN		6%	28%	0.00004041
	CPT		4%	3%	>0.05
	E		66%	49%	0.02284
	FOX		50%	35%	0.04212
	LEV		14%	7%	>0.05
	LZD		0%	6%	>0.05
	PG		84%	44%	0.000000008302
	T		21%	12%	>0.05
	TEC		11%	14%	>0.05

*mecA* gene positive rate			50.5%	35.3%	0.04212

ST	*S. aureus*	—	ST5^†^, ST7^†^, ST15^†^, ST25^†^, ST59^†^, ST398^†^, ST943^†^	ST25	—
	*S. epidermidis*	CC2	ST5, ST20, ST35, ST59, ST89, ST132, ST152, ST173, ST200, ST210, ST235, ST251, ST291, ST454	ST4, ST6, ST17, ST59, ST200, ST210, ST218, ST344, ST374,	—
		Minor CCs	ST192, ST247	ST325	—
		Other CCs	ST249	ST337, ST788, ST826, ST878, ST916	—
		New ST	ST840^*∗*^, ST841^*∗*^, ST842^*∗*^	—	—

^*∗*^New MLST type; ^†^data was cited from published work [9]. The same species and same MLST types are labeled in bold. HP, hospital personnel; FTS, frequently touched surface; C, chloramphenicol; CD, clindamycin; CN, gentamicin; CPT, ceftaroline; E, erythromycin; FOX, cefoxitin; LEV, levofloxacin; LZD, linezolid; PG, penicillin; T, tetracycline; TEC, teicoplanin; CC, clonal complex.

## Data Availability

The data generated or analyzed during this study are included within the article and supplemented materials.

## Supplementary Materials

Supplementary Materials The following supporting information can be downloaded at XX. Table S1. Antimicrobial susceptibility and molecular characterization of staphylococci that were recovered from hospital personnel; Table S2. Antimicrobial susceptibility and molecular characterization of staphylococci that were recovered from frequently touched surfaces.

## References

[1] Hu F., Zhu D., Wang F., Wang M. (2018). Current status and trends of antibacterial resistance in China. *Clinical Infectious Diseases*.

[2] Zhu Q., Yue Y., Zhu L. (2018). Epidemiology and microbiology of Gram-positive bloodstream infections in a tertiary-care hospital in Beijing, China: a 6-year retrospective study. *Antimicrobial Resistance and Infection Control*.

[3] Tian Y., Li T., Zhu Y., Wang B., Zou X., Li M. (2014). Mechanisms of linezolid resistance in staphylococci and enterococci isolated from two teaching hospitals in Shanghai, China. *BMC Microbiology*.

[4] Lin D., Ou Q., Lin J., Peng Y., Yao Z. (2017). A meta-analysis of the rates of *Staphylococcus aureus* and methicillin-resistant *S aureus* contamination on the surfaces of environmental objects that health care workers frequently touch. *American Journal of Infection Control*.

[5] Boyce J. M. (2007). Environmental contamination makes an important contribution to hospital infection. *Journal of Hospital Infection*.

[6] Lu P.-L., Siu L. K., Chen T.-C. (2009). Methicillin-resistant *Staphylococcus aureus* and *Acinetobacter baumanniion* computer interface surfaces of hospital wards and association with clinical isolates. *BMC Infectious Diseases*.

[7] Becker K., Heilmann C., Peters G. (2014). Coagulase-negative staphylococci. *Clinical Microbiology Reviews*.

[8] Xu Z., Shah H. N., Misra R. (2018). The prevalence, antibiotic resistance and *mecA* characterization of coagulase negative staphylococci recovered from non-healthcare settings in London, UK. *Antimicrobial Resistance and Infection Control*.

[9] Xu Z., Li X., Tian D. (2020). Molecular characterization of methicillin-resistant and-susceptible *Staphylococcus aureus* recovered from hospital personnel. *Journal of Medical Microbiology*.

[10] Wayne P. A. (2017). *CLSI Performance Standard of Antimicrobial Susceptibility Testing*.

[11] Uehara Y. (2022). Current status of staphylococcal cassette chromosome mec (SCCmec). *Antibiotics*.

[12] Kondo Y., Ito T., Ma X. X. (2007). Combination of multiplex PCRs for staphylococcal cassette chromosome mec type assignment: rapid identification system for *mec*, *ccr*, and major differences in junkyard regions. *Antimicrobial Agents and Chemotherapy*.

[13] Enright M. C., Day N. P. J., Davies C. E., Peacock S. J., Spratt B. G. (2000). Multilocus sequence typing for characterization of methicillin-resistant and methicillin-susceptible clones of *Staphylococcus aureus*. *Journal of Clinical Microbiology*.

[14] Thomas J. C., Vargas M. R., Miragaia M., Peacock S. J., Archer G. L., Enright M. C. (2007). Improved multilocus sequence typing scheme for Staphylococcus epidermidis. *Journal of Clinical Microbiology*.

[15] Morgenstern M., Erichsen C., Hackl S. (2016). Antibiotic resistance of commensal *Staphylococcus aureus* and coagulase-negative staphylococci in an international cohort of surgeons: a prospective point-prevalence study. *PLoS One*.

[16] Becker K., Both A., Weißelberg S., Heilmann C., Rohde H. (2020). Emergence of coagulase-negative staphylococci. *Expert Review of Anti-infective Therapy*.

[17] Pinho M. G., de Lencastre H., Tomasz A. (2001). An acquired and a native penicillin-binding protein cooperate in building the cell wall of drug-resistant staphylococci. *Proceedings of the National Academy of Sciences*.

[18] Heilmann C., Ziebuhr W., Becker K. (2019). Are coagulase-negative staphylococci virulent?. *Clinical Microbiology and Infections*.

[19] Oliveira D. C., Milheiriço C., de Lencastre H. (2006). Redefining a structural variant of staphylococcal cassette chromosome mec, SCCmec type VI. *Antimicrobial Agents and Chemotherapy*.

[20] Oliveira D. C., Tomasz A., de Lencastre H. (2002). Secrets of success of a human pathogen: molecular evolution of pandemic clones of meticillin-resistant *Staphylococcus aureus*. *The Lancet Infectious Diseases*.

[21] Du X., Zhu Y., Song Y. (2013). Molecular analysis of Staphylococcus epidermidis strains isolated from community and hospital environments in China. *PLoS One*.

[22] Xu Z., Misra R., Jamrozy D. (2018). Whole genome sequence and comparative genomics analysis of multi-drug resistant environmental *Staphylococcus epidermidis* ST59. *G3 (Bethesda, Md.)*.

[23] Liu Y., Chen L., Xu Z. (2022). Molecular Characterization of Staphylococci Recovered from Hospital Personnel and Frequently Touched Items in Tianjin, China.

