# Supplementary material for: Molecular Characterization of Staphylococci Recovered from Hospital Personnel and Frequently Touched Surfaces in Tianjin, China
Source: Can J Infect Dis Med Microbiol. 2022 Aug 10;2022:1061387. doi: 10.1155/2022/1061387 (PMC9385319; doi:10.1155/2022/1061387)
Supplement: Supplementary Materials — The following supporting information can be downloaded at XX. Table S1. Antimicrobial susceptibility and molecular characterization of staphylococci that were recovered from hospital personnel; Table S2. Antimicrobial susceptibility and molecular characterization of staphylococci that were recovered from frequently touched surfaces. [file 1061387.f1.zip › Supplementary Table S1.pdf]

Table S1 Antimicrobial susceptibility and molecular characterization of staphylococci that were recovered from hospital personnel

No.	sites	Dept	ID	C	CD	CN	CPT	E	FOX	LEV	LZD	PG	T	TEC	*mecA*	SCC*mec*	MLST
1	H	OT	*S.cohnii*	S	R	S	S	R	R	S	S	R	S	I	+	A/1	-
2	NC	TS	*S.epidermidis*	S	I	S	S	I	R	S	S	R	S	I	+	I	59
3	NC	IM	*S.epidermidis*	S	R	S	S	I	R	S	S	R	S	S	+	I	59
4	NC	DM	*S.epidermidis*	S	I	I	S	R	R	R	S	R	S	I	+	I	89
5	H	CM	*S.epidermidis*	S	S	S	S	R	R	S	S	R	R	R	+	I	247
6	H	CM	*S.epidermidis*	R	S	R	S	R	R	R	S	R	S	I	+	I	291
7	NC	DM	*S.epidermidis*	R	S	I	S	R	R	I	S	R	S	S	+	I	840*
8	H	RH	*S.epidermidis*	R	I	S	S	R	R	S	S	R	S	I	+	II	200
9	NC	OT	*S.epidermidis*	R	S	S	S	R	R	R	S	R	S	I	+	II	251
10	NC	ER	*S.epidermidis*	S	I	S	S	R	R	S	S	R	S	S	+	IV	35
11	NC	ER	*S.epidermidis*	S	R	S	S	I	R	S	S	R	S	I	+	IV	59
12	H	ER	*S.epidermidis*	S	R	S	S	I	R	S	S	R	S	I	+	IV	59
13	NC	OT	*S.epidermidis*	S	I	S	S	S	R	S	S	R	S	I	+	IV	59
14	H	RH	*S.epidermidis*	S	I	S	S	S	R	S	S	R	S	I	+	IV	59
15	NC	ER	*S.epidermidis*	S	S	S	S	S	R	R	S	R	S	R	+	IV	89
16	NC	ER	*S.epidermidis*	S	S	S	S	R	R	R	S	R	S	I	+	V	20
17	H	TS	*S.epidermidis*	S	I	S	S	R	R	S	S	R	S	I	+	V	35
18	NC	ER	*S.epidermidis*	S	S	I	S	R	R	S	S	R	S	S	+	V	35
19	NC	CM	*S.epidermidis*	S	I	S	S	R	R	R	S	R	S	I	+	V	89
20	NC	ER	*S.epidermidis*	S	S	S	S	S	R	S	S	R	S	I	+	V	89
21	NC	ER	*S.epidermidis*	S	R	S	S	R	R	S	S	R	S	S	+	V	89
22	NC	ER	*S.epidermidis*	S	R	S	S	R	R	R	S	R	R	S	+	V	89

23	NC	ER	*S.epidermidis*	S	S	S	S	S	R	R	S	R	S	S	+	V	152
24	NC	ER	*S.epidermidis*	S	R	S	S	R	R	S	S	R	S	I	+	V	192
25	NC	ER	*S.epidermidis*	S	S	S	S	R	R	S	S	R	S	I	+	V	192
26	NC	DM	*S.epidermidis*	S	S	S	S	R	R	R	S	R	S	I	+	V	210
27	NC	ER	*S.epidermidis*	S	R	S	S	R	R	R	S	R	S	I	+	V	210
28	H	ED	*S.epidermidis*	S	I	S	S	I	R	S	S	R	S	I	+	V	454
29	H	ED	*S.epidermidis*	S	S	S	S	R	R	S	S	R	S	I	+	V	841*
30	NC	IM	*S.epidermidis*	S	S	S	S	R	R	S	S	R	S	I	+	V	842*
31	NC	ER	*S.epidermidis*	R	I	R	S	S	R	R	S	R	S	I	+	IX	5
32	H	ER	*S.epidermidis*	R	S	S	S	S	R	I	S	R	S	I	+	IX	235
33	H	OT	*S.epidermidis*	S	R	S	R	R	R	S	S	R	S	S	+	A/1	249
34	NC	DM	*S.epidermidis*	S	I	R	S	R	R	S	S	R	R	S	+	B/5	59
35	NC	DM	*S.epidermidis*	R	S	S	S	I	R	R	S	R	R	I	+	B/5	235
36	NC	ER	*S.epidermidis*	I	R	S	I	I	R	S	S	R	S	S	+	C/2	59
37	NC	ER	*S.epidermidis*	R	S	S	S	I	R	I	S	R	S	I	+	C/2	235
38	NC	ER	*S.epidermidis*	R	R	R	S	I	R	S	S	R	R	I	+	SCC	59
39	NC	OT	*S.epidermidis*	S	S	S	S	R	R	S	S	R	S	I	+	SCC	132
40	NC	OT	*S.epidermdis*	R	I	S	S	R	R	S	S	R	S	R	+	Pseudo (ψ) SCC	173
41	H	ED	*S.hominis*	S	R	S	S	R	R	S	S	R	S	S	+	A/1	-
42	H	MEC	*S.hominis*	S	R	S	R	R	R	S	S	S	R	I	+	A/5	-
43	H	TS	*S.hominis*	S	I	S	S	S	R	S	S	R	S	S	+	I	-
44	H	ED	*S.hominis*	R	I	S	R	R	R	S	S	S	S	S	+	I	-
45	H	OT	*S.hominis*	R	R	S	I	R	R	S	S	R	S	S	+	I	-
46	H	MEC	*S.hominis*	S	I	S	S	R	R	S	S	S	S	S	+	II	-

47	H	MEC	*S.hominis*	S	S	S	S	R	R	S	S	S	R	S	+	II	-
48	H	MEC	*S.hominis*	S	S	S	S	R	R	S	S	S	S	S	+	II	-
49	H	MEC	*S.hominis*	R	S	S	S	R	R	I	S	S	R	S	+	II	-
50	H	MEC	*S.hominis*	S	S	S	S	R	R	S	S	R	S	S	+	II	-
51	H	OT	*S.hominis*	R	S	S	S	R	R	S	S	R	R	S	+	II	-
52	NC	OT	*S.hominis*	S	I	S	S	R	R	S	S	S	S	S	+	II	-
53	NC	MEC	*S.hominis*	S	I	S	S	R	R	S	S	R	R	S	+	V	-
54	H	ED	*S.hominis*	S	I	S	S	R	R	S	S	R	R	S	+	A/5	-
55	H	OT	*S.hominis*	S	I	S	S	R	R	R	S	S	S	S	+	SCC	-
56	H	TS	*S.capitis*	S	S	S	S	S	S	S	S	R	S	I	-	-	-
57	H	DM	*S.cohnii*	S	R	R	S	R	R	S	S	R	I	I	-	-	-
58	H	CM	*S.epidermidis*	S	S	S	S	R	R	S	S	R	R	S	-	-	-
59	NC	CM	*S.epidermidis*	S	I	S	S	S	S	R	S	R	S	R	-	-	-
60	NC	CM	*S.epidermidis*	S	I	S	S	R	S	S	S	R	S	R	-	-	-
61	NC	CM	*S.epidermidis*	S	S	S	S	R	S	S	S	S	S	I	-	-	-
62	H	TS	*S.epidermidis*	I	R	S	I	R	S	S	S	R	S	S	-	-	-
63	H	DM	*S.epidermidis*	S	I	S	S	R	S	S	S	R	S	I	-	-	-
64	H	ED	*S.epidermidis*	S	R	S	R	R	S	S	S	S	S	S	-	-	-
65	H	ED	*S.epidermidis*	S	S	S	S	R	S	S	S	R	S	R	-	-	-
66	H	ED	*S.epidermidis*	S	S	S	S	I	R	S	S	R	R	S	-	-	-
67	H	ED	*S.epidermidis*	S	S	S	S	R	S	S	S	R	S	I	-	-	-
68	H	ER	*S.epidermidis*	S	R	S	S	R	S	S	S	R	S	S	-	-	-
69	H	ER	*S.epidermidis*	S	S	S	S	S	S	S	S	R	S	I	-	-	-
70	H	ER	*S.epidermidis*	S	I	S	S	R	S	S	S	R	S	R	-	-	-

71	NC	ER	*S.epidermidis*	S	I	S	S	I	S	S	S	S	S	S	-	-	-
72	H	ER	*S.epidermidis*	S	R	S	S	R	S	S	S	R	S	I	-	-	-
73	NC	PT	*S.epidermidis*	S	S	S	S	I	S	S	S	R	S	R	-	-	-
74	NC	PT	*S.epidermidis*	S	S	S	S	I	S	S	S	R	S	R	-	-	-
75	NC	PT	*S.epidermidis*	S	I	S	S	S	S	S	S	R	S	I	-	-	-
76	NC	PT	*S.epidermidis*	S	S	S	S	I	S	S	S	R	S	S	-	-	-
77	NC	PT	*S.epidermidis*	S	S	S	S	I	R	S	S	R	S	S	-	-	-
78	NC	PT	*S.epidermidis*	S	R	S	S	I	S	S	S	R	S	R	-	-	-
79	NC	PT	*S.epidermidis*	S	S	S	S	R	S	S	S	R	S	S	-	-	-
80	H	PT	*S.epidermidis*	S	S	S	S	I	S	S	S	R	S	I	-	-	-
81	NC	PT	*S.epidermidis*	S	S	S	S	S	S	S	S	R	S	S	-	-	-
82	NC	RH	*S.epidermidis*	S	S	S	S	S	S	S	S	R	R	S	-	-	-
83	NC	RH	*S.epidermidis*	S	S	S	S	I	S	S	S	S	S	S	-	-	-
84	H	RH	*S.epidermidis*	S	I	S	S	I	R	S	S	R	S	I	-	-	-
85	H	RH	*S.epidermidis*	S	I	S	S	S	S	R	S	R	S	S	-	-	-
86	H	RH	*S.epidermidis*	S	S	S	S	S	S	S	S	R	S	S	-	-	-
87	NC	RH	*S.epidermidis*	S	S	S	S	R	S	S	S	S	S	I	-	-	-
88	NC	RH	*S.epidermidis*	S	S	S	S	S	S	S	S	R	R	I	-	-	-
89	NC	RH	*S.epidermidis*	S	I	S	S	R	S	S	S	R	R	R	-	-	-
90	H	RH	*S.epidermidis*	S	I	S	S	R	S	S	S	R	R	R	-	-	-
91	H	CM	*S.hominis*	S	I	S	S	R	S	S	S	R	S	S	-	-	-
92	H	ED	*S.hominis*	S	S	S	S	R	R	S	S	R	S	S	-	-	-
93	H	ER	*S.hominis*	S	I	S	S	R	R	S	S	S	R	S	-	-	-

94	H	PT	*S.hominis*	R	I	S	S	R	R	S	S	S	R	S	-	-	-
95	H	PT	*S.hominis*	R	I	S	S	R	R	S	S	S	R	S	-	-	-
96	NC	RH	*S.hominis*	S	S	S	S	R	R	S	S	R	S	S	-	-	-
97	H	RH	*S.hominis*	S	S	S	S	R	R	S	S	S	S	S	-	-	-
98	H	RH	*S.hominis*	S	S	S	S	R	R	S	S	R	R	S	-	-	-
99	H	RH	*S.hominis*	S	S	S	S	R	R	S	S	R	R	S	-	-	-
100	NC	RH	*S.hominis*	S	S	S	S	R	S	S	S	R	S	S	-	-	-
101	NC	RH	*S.hominis*	S	S	S	S	R	S	S	S	R	S	S	-	-	-
102	H	RH	*S.hominis*	S	S	S	S	R	S	S	S	R	S	S	-	-	-
103	H	RH	*S.pasteuri*	S	S	S	S	R	S	S	S	R	S	S	-	-	-
104	H	ED	*S.warneri*	S	I	S	S	R	S	S	S	R	S	I	-	-	-
105	H	ER	*S.warneri*	S	S	R	S	I	S	S	S	R	S	S	-	-	-
106	H	DM	*S.warneri*	S	I	S	S	R	R	S	S	R	R	I	-	-	-
107	H	DM	*S.warneri*	I	I	I	S	R	S	S	S	R	S	S	-	-	-
108	H	RH	*S.warneri*	S	I	S	S	R	S	S	S	R	S	I	-	-	-
109	H	RH	*S.warneri*	S	S	S	S	R	R	S	S	R	S	I	-	-	-

Note: CM: Chinese medicine department, DM: dermatology department, ED: endocrine department, ER: emergency room, IM: ultrasonic department, MEC: medical examine centre, OT: orthopaedics department, PT: proctology department, RH: rehabilitation department, TS: thoracic surgery department; * new MLST type； C: chloramphenicol, CD: clindamycin, CN: gentamicin, CPT: ceftaroline, E: erythromycin, FOX: cefoxitin, LEV: levofloxacin, LZD: linezolid, PG: penicillin, T: tetracycline, TEC: teicoplanin
