# Supplementary material for: Molecular Characterization of Staphylococci Recovered from Hospital Personnel and Frequently Touched Surfaces in Tianjin, China
Source: Can J Infect Dis Med Microbiol. 2022 Aug 10;2022:1061387. doi: 10.1155/2022/1061387 (PMC9385319; doi:10.1155/2022/1061387)
Supplement: Supplementary Materials — The following supporting information can be downloaded at XX. Table S1. Antimicrobial susceptibility and molecular characterization of staphylococci that were recovered from hospital personnel; Table S2. Antimicrobial susceptibility and molecular characterization of staphylococci that were recovered from frequently touched surfaces. [file 1061387.f1.zip › Supplementary Table S2.pdf]

Table S2 Antimicrobial susceptibility and molecular characterization of staphylococci that were recovered from frequently touched surfaces 1

No	dept	sites	ID	C	CD	CN	CPT	E	FOX	LEV	LZD	PG	T	TEC	*mecA*	SCCmec	MLST
1	CT	registration machine	*S. capitis*	I	S	S	I	I	R	R	R	S	R	S	+	SCC	-
2	OC	door handle	*S. epidermidis*	R	I	R	S	R	R	I	S	R	S	S	+	II	17
3	OC	door handle	*S. epidermidis*	S	S	R	S	I	R	R	S	S	S	S	+	IV	4
4	ND	door handle	*S. epidermidis*	I	S	S	S	I	R	S	S	S	S	S	+	IV	4
5	LG	door handle	*S. epidermidis*	S	I	R	I	R	R	S	S	R	S	R	+	IV	6
6	ENT	door handle	*S. epidermidis*	S	S	R	S	R	R	S	S	R	S	R	+	IV	17
7	ND	door handle	*S. epidermidis*	S	R	S	S	R	R	S	S	S	S	S	+	IV	59
8	OC	door handle	*S. epidermidis*	S	S	S	S	S	R	S	S	S	S	S	+	IV	344
9	DM	door handle	*S. epidermidis*	S	S	R	S	R	R	R	S	R	S	S	+	V	210
10	SD	door handle	*S. epidermidis*	S	S	S	S	R	R	R	S	R	S	I	+	V	210
11	ND	door handle	*S. epidermidis*	S	S	S	S	R	R	S	S	S	S	S	+	V	337
12	OC	door handle	*S. epidermidis*	S	S	R	S	S	R	S	S	R	S	S	+	A/1	916
13	CL	report machine	*S. epidermidis*	S	S	S	S	R	R	S	S	R	S	I	+	B/5	374
14	DM	door handle	*S. epidermidis*	S	I	R	S	R	R	S	S	R	S	I	+	C/1	878
15	CT	registration machine	*S. epidermidis*	S	S	S	S	R	R	S	S	R	S	S	+	C/2	218
16	ENT	door handle	*S. epidermidis*	S	S	S	S	R	R	S	S	R	S	S	+	C/2	826
17	SD	door handle	*S. epidermidis*	S	I	S	S	S	R	S	S	R	S	I	+	SCC	200
18	LG	door handle	*S. epidermidis*	S	S	S	S	S	R	S	S	R	S	S	+	SCC	325
19	IM	door handle	*S. epidermidis*	S	S	S	S	R	R	S	S	R	S	S	+	SCC	788
20	DM	checking bed	*S. haemolyticus*	S	S	S	S	R	R	I	S	S	S	S	+	II	-
21	OT	door handle	*S. haemolyticus*	R	R	R	I	R	R	I	S	R	R	I	+	V	-

22	ER	door handle	*S. hominis*	S	S	S	S	S	R	S	R	S	S	S	+	A/5	-
23	OD	call button	*S. hominis*	S	S	R	S	I	R	S	R	R	S	S	+	I	-
24	LG	checking bed	*S. hominis*	S	S	S	S	S	R	S	S	S	S	S	+	II	-
25	LG	checking bed	*S. hominis*	S	S	S	S	S	R	S	S	S	S	S	+	Pseudo (ψ) SCCmec	
26	PD	water tap	*S. hominis*	S	S	S	S	S	R	S	S	S	S	S	+	Pseudo (ψ) SCC	
27	ED	door handle	*S. pasteuri*	S	S	S	S	R	R	S	S	R	S	S	+	IV	-
28	ER	registration machine	*S. saprophyticus*	R	S	S	S	I	R	S	S	R	R	S	+	V	-
29	DG	checking bed	*S. warneri*	S	R	R	I	R	R	S	S	I	S	I	+	V	-
30	ED	checking bed	*S. warneri*	S	S	S	S	I	R	S	S	S	S	S	+	VI	-
31	ED	door handle	*S. aureus*	I	I	S	R	R	S	S	S	R	S	R	-	-	-
32	CT	door handle	*S. capitis*	S	I	S	S	S	S	S	S	S	S	S	-	-	-
33	CT	door handle	*S. capitis*	S	I	S	S	I	S	S	S	S	S	S	-	-	-
34	DM	laser machine	*S. capitis*	S	I	S	S	I	S	S	S	R	S	R	-	-	-
35	ED	checking bed	*S. capitis*	S	R	R	S	S	S	S	S	R	S	R	-	-	-
36	ED	door handle	*S. capitis*	S	I	S	S	R	S	S	S	R	R	I	-	-	-
37	ENT	door handle	*S. capitis*	I	R	R	R	I	S	R	R	R	R	S	-	-	-
38	IM	door handle	*S. capitis*	S	S	S	S	S	S	S	S	S	S	S	-	-	-
39	IM	door handle	*S. capitis*	S	I	S	S	S	S	S	S	R	S	S	-	-	-
40	OT	call button	*S. capitis*	S	S	S	S	S	S	S	S	S	S	S	-	-	-
41	OT	call button	*S. capitis*	S	S	S	S	S	S	S	S	S	S	S	-	-	-
42	AN	checking bed	*S. capitis*	S	S	S	S	I	S	S	S	R	S	S	-	-	-
43	MD	door handle	*S. capitis*	S	S	S	S	I	S	S	S	R	S	S	-	-	-
44	GT	checking bed	*S. cohnii*	S	I	S	S	R	S	S	S	R	S	I	-	-	-
45	GT	water tap	*S. cohnii*	S	S	S	S	R	S	S	S	S	S	S	-	-	-

46	ER	registration machine	*S. cohnii*	S	R	S	S	R	S	S	S	R	S	S	-	-	-
47	IM	door handle	*S. cohnii*	I	R	I	I	R	S	I	S	R	S	I	-	-	-
48	IM	door handle	*S. cohnii*	S	R	I	S	R	S	S	S	R	S	I	-	-	-
49	ENT	door handle	*S. cohnii*	S	S	S	S	R	S	S	S	S	S	S	-	-	-
50	CT	water tap	*S. epidermidis*	S	I	S	S	S	S	S	S	R	S	I	-	-	-
51	CT	water tap	*S. epidermidis*	S	I	S	S	S	S	S	S	R	S	I	-	-	-
52	GT	door handle	*S. epidermidis*	S	S	S	S	S	S	S	S	S	S	S	-	-	-
53	LG	door handle	*S. epidermidis*	S	S	S	S	S	S	S	S	R	S	S	-	-	-
54	OT	call button	*S. epidermidis*	S	S	S	S	S	S	S	S	S	S	I	-	-	-
55	PD	door handle	*S. epidermidis*	S	R	S	S	R	S	S	S	S	S	I	-	-	-
56	AR	checking bed	*S. epidermidis*	S	S	S	S	S	S	S	S	R	S	I	-	-	-
57	AR	water tap	*S. epidermidis*	R	R	S	S	R	S	S	S	S	S	S	-	-	-
58	ENT	door handle	*S. epidermidis*	R	S	S	S	R	S	R	S	R	S	I	-	-	-
59	DM	water tap	*S. equorum*	R	S	S	S	I	S	S	S	S	R	I	-	-	-
60	IM	water tap	*S. equorum*	S	S	S	S	R	S	S	S	S	S	S	-	-	-
61	OT	door handle	*S. haemolyticus*	S	I	R	I	R	S	S	S	S	R	R	-	-	-
62	PD	water tap	*S. haemolyticus*	S	R	S	S	R	S	S	S	S	S	I	-	-	-
63	MD	checking bed	*S. haemolyticus*	S	R	R	R	I	S	S	S	S	S	R	-	-	-
64	NS	checking bed	*S. haemolyticus*	S	S	S	I	S	S	S	R	S	S	R	-	-	-
65	CT	door handle	*S. hominis*	S	S	S	S	S	S	S	S	S	S	S	-	-	-
66	GT	water tap	*S. hominis*	R	I	R	S	R	S	S	S	S	S	R	-	-	-
67	IM	door handle	*S. hominis*	S	R	R	S	R	S	S	S	S	R	I	-	-	-
68	OT	call button	*S. hominis*	S	S	S	S	S	S	S	S	S	S	S	-	-	-
69	OT	call button	*S. hominis*	S	S	S	S	S	S	S	S	S	S	S	-	-	-

70	PD	door handle	*S. hominis*	R	R	S	S	R	S	S	S	S	R	S	-	-	-
71	SD	door handle	*S. hominis*	S	S	S	S	R	S	S	S	R	S	I	-	-	-
72	NS	checking bed	*S. hominis*	S	S	S	S	R	S	S	S	S	S	S	-	-	-
73	LG	checking bed	*S. pasteuri*	S	S	S	S	S	S	S	S	S	S	S	-	-	-
74	IM	door handle	*S. pettenkoferi*	S	S	S	S	S	S	S	S	S	S	S	-	-	-
75	PD	door handle	*S. saprophyticus*	S	I	S	S	I	S	S	S	R	R	I	-	-	-
76	SD	checking equipment	*S. saprophyticus*	S	I	S	S	I	S	S	S	R	S	I		-	-
77	OT	call button	*S. succinus*	S	S	R	S	S	S	S	S	S	S	I	-	-	-
78	CM	door handle	*S. warneri*	R	S	R	S	R	S	S	S	S	S	I	-	-	-
79	CM	door handle	*S. warneri*	R	S	R	S	R	S	S	S	S	S	R	-	-	-
80	CM	door handle	*S. warneri*	R	R	R	S	R	S	S	S	S	S	I	-	-	-
81	CM	door handle	*S. warneri*	R	S	R	S	R	S	S	S	S	S	I	-	-	-
82	CT	water tap	*S. warneri*	S	S	S	S	S	S	S	S	S	S	R	-	-	-
83	CT	water tap	*S. warneri*	S	S	S	S	S	S	S	S	S	I	R	-	-	-
84	ENT	door handle	*S. warneri*	S	I	R	S	R	S	S	S	S	S	I	-	-	-
85	PD	water tap	*S. warneri*	S	S	R	S	R	S	S	S	S	S	I	-	-	-
86	NS	water tap	*S. warneri*	S	I	R	S	R	S	S	S	S	S	I	-	-	-

Note: AR: anorectal surgery.CL: clinical lab, CM: Chinese medicine department, CT: Chest disease department, DG: digestion medicine, DM: dermatology department, ED: endocrine department, ENT: E.N.T. department, ER: emergency room, IM: ultrasonic department, LG: liver and gall surgical department, ND: neurology department, OC: oncology department, OT: orthopaedics department, PD: pediatric department, SD: stomatology department, NS: neurosurgery; GT: gastroenterology, MD: medicine department; C: chloramphenicol, CD: clindamycin, CN: gentamicin, CPT: ceftaroline, E: erythromycin, FOX: cefoxitin, LEV: levofloxacin, LZD: linezolid, PG: penicillin, T: tetracycline, TEC: teicoplanin
